# NR2 subunits and NMDA receptors on lamina II inhibitory and excitatory interneurons of the mouse dorsal horn

**DOI:** 10.1186/1744-8069-6-26

**Published:** 2010-05-06

**Authors:** Hiroaki Shiokawa, Edward J Kaftan, Amy B MacDermott, Chi-Kun Tong

**Affiliations:** 1 Department of Physiology and Cellular Biophysics, Columbia University, New York, New York 10032, USA; 2 Department of Neuroscience, Columbia University, New York, New York 10032, USA; 3 Pain Research, Discovery Neuroscience, Wyeth Research, Princeton, NJ 08534, USA

## Abstract

**Background:**

NMDA receptors expressed by spinal cord neurons in the superficial dorsal horn are involved in the development of chronic pain associated with inflammation and nerve injury. The superficial dorsal horn has a complex and still poorly understood circuitry that is mainly populated by inhibitory and excitatory interneurons. Little is known about how NMDA receptor subunit composition, and therefore pharmacology and voltage dependence, varies with neuronal cell type. NMDA receptors are typically composed of two NR1 subunits and two of four NR2 subunits, NR2A-2D. We took advantage of the differences in Mg^2+ ^sensitivity of the NMDA receptor subtypes together with subtype preferring antagonists to identify the NR2 subunit composition of NMDA receptors expressed on lamina II inhibitory and excitatory interneurons. To distinguish between excitatory and inhibitory interneurons, we used transgenic mice expressing enhanced green fluorescent protein driven by the GAD67 promoter.

**Results:**

Analysis of conductance ratio and selective antagonists showed that lamina II GABAergic interneurons express both the NR2A/B containing Mg^2+ ^sensitive receptors and the NR2C/D containing NMDA receptors with less Mg^2+ ^sensitivity. In contrast, excitatory lamina II interneurons express primarily NR2A/B containing receptors. Despite this clear difference in NMDA receptor subunit expression in the two neuronal populations, focally stimulated synaptic input is mediated exclusively by NR2A and 2B containing receptors in both neuronal populations.

**Conclusions:**

Stronger expression of NMDA receptors with NR2C/D subunits by inhibitory interneurons compared to excitatory interneurons may provide a mechanism to selectively increase activity of inhibitory neurons during intense excitatory drive that can provide inhibitory feedback.

## Background

The large majority of neurons in the superficial dorsal horn are local circuitry interneurons, including both excitatory and inhibitory interneurons [1]. Inhibition controls the flow of sensory input through mono and polysynaptic excitatory pathways to dorsal horn projection neurons and thus to higher brain centers. Sensory inputs include the modalities of pain, itch, temperature, and some mechanosensation. A good example of the interplay between excitation and inhibition in the dorsal horn is revealed by the disinhibition that sometimes accompanies peripheral nerve injury. This disinhibition produces allodynia, a painful response to a stimulus that is usually non-painful or innocuous. The disinhibition driving the behavioral phenomenon of allodynia can be mimicked by intrathecal administration of the GABA_A _receptor antagonist, bicuculline, or the glycine receptor antagonist strychnine [2-5]. These behavioral changes associated with disinhibition are consistent with a chronic pain condition.

NMDA receptors are expressed at glutamatergic synapses throughout the superficial dorsal horn and have been implicated in driving the new excitatory activity that accompanies disinhibition. For example, disinhibition *in vitro *strongly enhances low-threshold (Aβ fiber) driven polysynaptic input onto lamina I projection neurons relayed by excitatory interneurons [4,6,7]. In the presence of the NMDA receptor antagonist D-APV, this recruitment of polysynaptic Aβ fiber input onto lamina II interneurons and lamina I projection neurons is abolished [4,6,8]. *In vivo *studies also show that D-APV prevents disinhibition-induced allodynia [3,5]. These results suggest that NMDA receptors expressed on dorsal horn interneurons, especially excitatory interneurons, are essential for the development of allodynia induced by disinhibition. It is also likely, however, that NMDA receptors expressed on inhibitory interneurons are important for modulating inhibitory control of excitatory pathways. Thus, identifying the functional properties of NMDA receptors expressed on inhibitory and excitatory interneurons is important not only for providing insight into the mechanisms regulating excitation and inhibition in the dorsal horn, but also for developing strategies for targeted clinical treatments.

Most commonly, NMDA receptors consist of two NR1 subunits and two NR2 subunits of which there are four types: NR2A, NR2B, NR2C,NR2D [9]. NMDA receptors with different NR2 subunits show diverse functional characteristics such as affinity of agonists and antagonists, receptor kinetic properties, channel conductances, and voltage dependent Mg^2+ ^sensitivity [10]. Receptors with NR2A or NR2B subunits show higher Mg^2+ ^sensitivity at negative membrane potentials than those with NR2C or NR2D subunits. Projection neurons in lamina I expressing NK1 receptors (NK1R+) express heterogeneous NMDA receptors including those that are NR2A/B like and NR2C/D like [11]. Lamina II neurons express different types of NMDA receptors at subsynaptic and extrasynaptic regions [12]. However, little is known about how the composition of NMDA subunits might differ between excitatory or inhibitory interneurons in lamina II.

In this study, we took advantage of a recently developed technique [11] to functionally identify different NMDA receptor subtypes with different Mg^2+ ^sensitivities expressed by inhibitory and excitatory interneurons in lamina II.

## Results

### Pre-identified interneurons in lamina II are tested with NMDA superfusion

Essentially all of the neurons in lamina II of the dorsal horn are interneurons. Lamina II can be distinguished from other laminae based on the translucent appearance of that cell layer under transmitted light. Seventy percent of the interneurons are excitatory and 30% are inhibitory [13]. To identify inhibitory neurons, we used GAD67 EGFP knock-in mice [14]. Double label experiments indicate that 30-70% of the GABAergic lamina II interneurons are co-labeled with EGFP [15,16]. EGFP negative cells are a mixture of excitatory interneurons and GABAergic interneurons that are not labeled by EGFP. Therefore about ~80-90% of EGFP negative cells are excitatory interneurons. Because all of the EGFP positive neurons are GABAergic interneurons, we used EGFP negative lamina II neurons as putative excitatory interneurons, even though there is a 10-20% 'contamination' with inhibitory interneurons.

**Figure 1:**
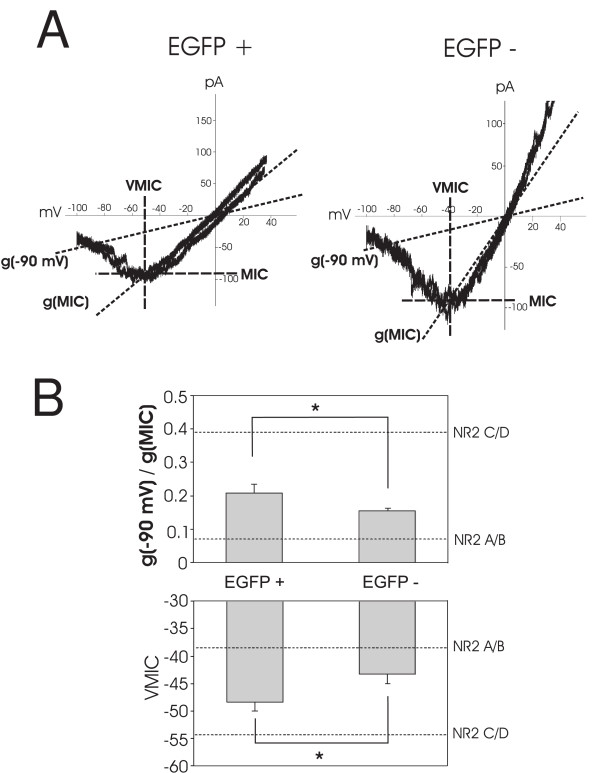
**Analysis of the voltage dependence of NMDA receptors expressed in EGFP+ and EGFP- cells in the presence of 100 μM Mg^2+^**. A, the current - voltage relationship recorded at the peak response to NMDA application in an EGFP+ and an EGFP- neuron. The maximal inward current (MIC) and voltage of maximal inward current (VMIC) are indicated by dashed lines. The conductance of NMDA receptors at -90 mV (g(-90 mV)) and conductance of maximal inward current (g(MIC)) are indicated by dotted lines. B, average g(-90 mV)/g(MIC) in EGFP+ and EGFP- interneurons (*P < 0.05, upper box) and the average VMIC in EGFP+ and EGFP- interneurons (*P < 0.05, lower box). Perforated lines indicate the conductance ratio of NR2A/B or NR2C/D receptor induced currents and VMIC derived from oocyte expression data of Kuner and Schoepfer [19].

NMDA currents were recorded under voltage clamp control from EGFP+ and EGFP- interneurons in spinal cord slices taken from postnatal day 14 (P14) to P21 mice. Bath application of NMDA (15 μM) generated inward currents at a holding potential of -70 mV. To evaluate the voltage-dependent Mg^2+ ^sensitivity of these receptors, triangle voltage ramps from -100 mV to +50 mV and back to -100 mV were applied at 20 second intervals before, during and after NMDA application [11]. The control current response to the voltage ramp was subtracted from NMDA receptor-mediated current ramps when the agonist response was at maximum (see Methods). The result was plotted as a function of command voltage as shown in Figure 1A. The voltage sensitivity of the NMDA current generated by the ascending ramp command was similar to that generated by the descending ramp command (Figure 1A). In addition, the current-voltage relationships of the NMDA induced currents had an average reversal potential of 1.4 ± 0.5 mV (n = 23), close to the predicted NMDA receptor reversal potential, indicating good voltage control.

Previously, we demonstrated a new method to identify subunit-associated characteristics of NMDA receptors expressed by neurons, including lamina I NK1R+ neurons in the spinal cord [11]. We identified NR2 subunit composition of NMDA receptors by focusing on the differential sensitivities to Mg^2+ ^inherent in NMDA receptors composed of different NR2 subunits. The voltage sensitivity of NMDA currents depends predominantly on the voltage dependence of Mg^2+ ^block of the receptors [17,18]. NMDA receptors containing NR2A or NR2B subunits show more negative slope conductance at negative membrane potentials than those containing NR2C or NR2D, meaning that NMDA receptors with NR2A or NR2B show higher Mg^2+ ^sensitivity at negative membrane potentials than those with NR2C or NR2D [17,19]. Because the measurable difference in voltage dependent Mg^2+ ^sensitivity among receptor types is enhanced when extracellular Mg^2+ ^concentration is low, 100 μM extracellular Mg^2+ ^was used throughout the current experiments.

**Figure 2:**
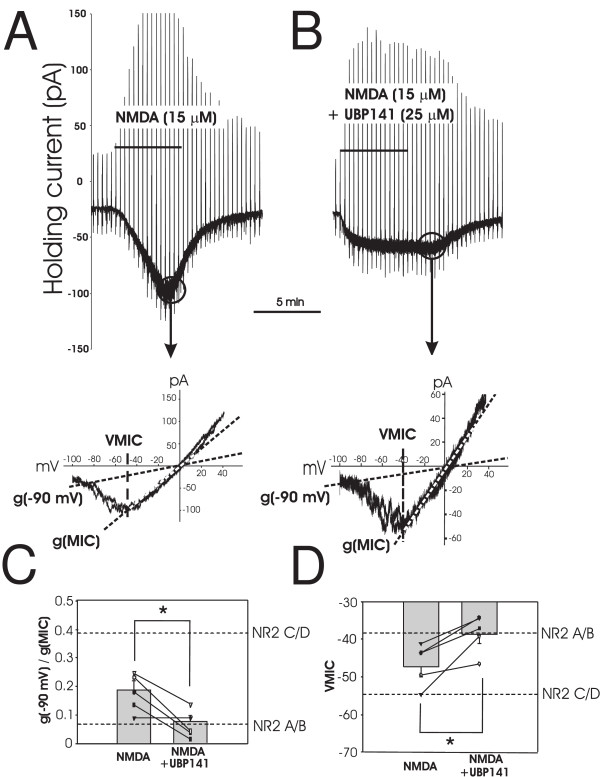
**Effect of NR2C/D preferring antagonist on NMDA induced current recorded from EGFP+ neurons**. A, NMDA induced inward current in an EGFP+ dorsal horn neuron. Current responses to voltage ramps during NMDA application are cut off at the peak of the response to allow direct comparison of the NMDA response to that in B. Current - voltage relationship obtained at the peak of the NMDA response is plotted below with the g(-90 mV) and g(MIC) indicated by dotted lines and VMIC indicated by dashed lines. B, Current responses to voltage ramps during co-application of 15 μM NMDA and 25 μM UBP141, an NR2C/D preferring antagonist, to the same cell as in A. Current - voltage relationship from the peak response is again plotted below with the g(-90 mV) and g(MIC) indicated by dotted lines and VMIC indicated by dashed lines. C shows both individual and average g(-90 mV)/g(MIC) values in the absence and presence of UBP141 (*P < 0.05). D indicates individual and average VMIC values in the absence and presence of UBP141 (*P < 0.05). For C and D, perforated lines indicate the conductance ratio of NR2A/B or NR2C/D receptor induced currents and VMIC derived from oocyte expression data of Kuner and Schoepfer [19].

**Figure 3:**
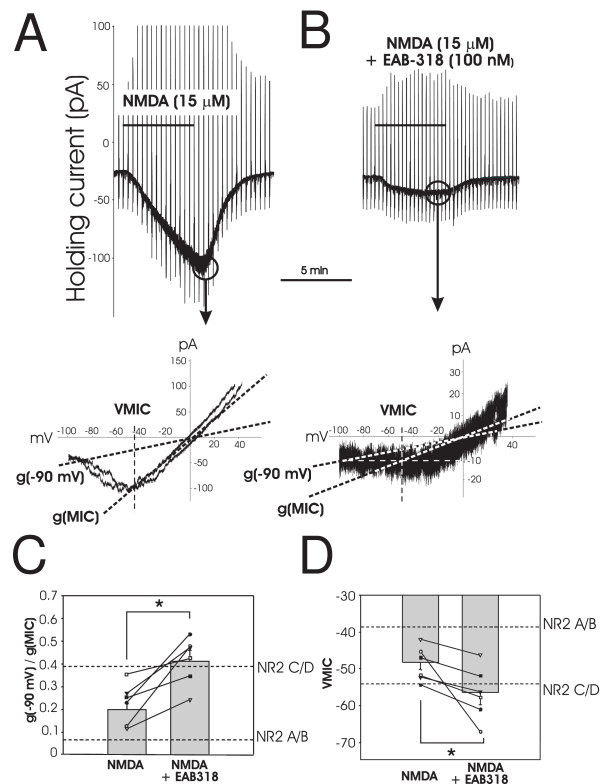
**Effect of NR2A/B preferring antagonist on NMDA induced current recorded from EGFP+ neurons**. A, NMDA induced inward current in an EGFP+ dorsal horn neuron. Current responses to voltage ramps during NMDA application are cut off at the peak of the response to allow direct comparison of the NMDA response to that in B. Current - voltage relationship obtained at the peak of the NMDA response is plotted below with the g(-90 mV) and g(MIC) indicated by dotted lines and VMIC indicated by dashed lines. B, current responses to voltage ramps during co-application of 15 μM NMDA and 100 nM EAB318, an NR2A/B selective antagonist, to the same cell as in A with the current -voltage relationship shown below. C, both individual and average g(-90 mV)/g(MIC) values in the absence and presence of EAB318 (*P < 0.05) are shown. D shows individual and average VMIC values in the absence and presence of EAB318 (*P < 0.05). For C and D, perforated lines indicate the conductance ratio of NR2A/B or NR2C/D receptor induced currents and VMIC derived from oocyte expression data of Kuner and Schoepfer [19].

**Figure 4:**
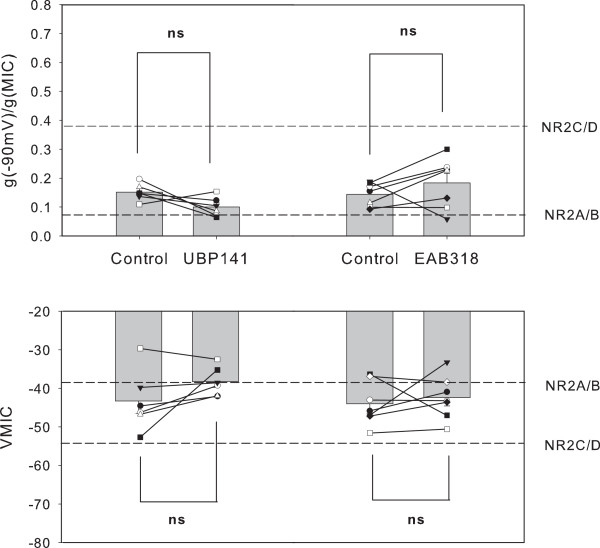
**No significant effect of NR2A/B or NR2C/D preferring antagonists on NMDA induced currents recorded from EGFP- neurons**. The g(-90 mV)/g(MIC) values and VMIC are plotted for individual neurons and average values in the absence and presence of 25 μM UBP141 or 100 nM EAB318. Neither UBP141 or EAB318 significantly changed g(-90 mV)/g(MIC) and VMIC values (ns; no significance). Perforated lines indicate the conductance ratio of NR2A/B or NR2C/D receptor induced currents and VMIC derived from oocyte expression data of Kuner and Schoepfer [19].

From the I-V curves recorded repeatedly during agonist application, we determined the voltage dependence of NMDA currents in a manner independent of absolute current amplitude [11]. The current at -90 mV (I(-90 mV)), the maximal inward current (MIC) and the membrane potential at which MIC occurs (VMIC) were measured as illustrated in Figure 1A for an EGFP+ and EGFP- neuron. The membrane conductance at -90 mV (g(-90 mV)) and MIC (g(MIC)) were calculated as shown in Figure 1A. We evaluated the voltage sensitivity of the agonist activated NMDA currents by calculating the ratio of g(-90 mV) to g(MIC) (conductance ratio) and by measuring VMIC. When we performed a similar analysis on NMDA receptor mediated currents recorded from oocytes containing NR1/NR2A or NR1/NR2B [19], we calculated g(-90 mV)/g(MIC) ratios of about 0.07 with VMIC between -37 and -40 mV. Conversely, NMDA receptors containing NR1/NR2C or NR1/NR2D had calculated ratios around 0.39 and VMICs around -52 to -57 mV [11,19]. These values are indicated by the perforated lines in Figures 1, 2, 3, 4, &5, for the purposes of comparison.

**Figure 5:**
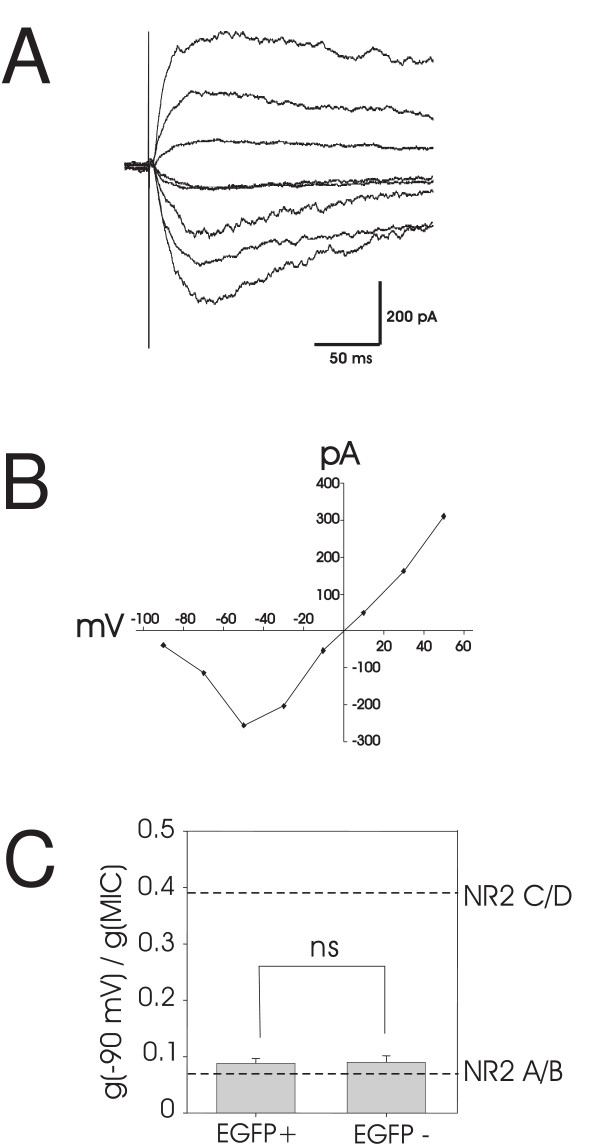
**Analysis of voltage dependent sensitivity of synaptic NMDA receptors to 100 μM Mg^2+ ^in lamina II interneurons**. A, Representative traces of focal stimulation evoked NMDA-EPSCs recorded at different holding potentials (-90 mV to +50 mV at 20 mV increment). B, Current - voltage relationship of NMDA EPSCs shown in A. The reversal potential for this example neuron is 0 mV. C shows that synaptic NMDA EPSCs from EGFP+ (n = 18) and EGFP- (n = 19) neurons have similar g(-90 mV)/g(MIC) that are comparable to the value of NMDA receptors with NR2A/B subunits. Perforated lines indicate the conductance ratio of NR2A/B or NR2C/D receptor induced currents derived from oocyte expression data.

Figure 1B shows the mean conductance ratio when NMDA evoked currents were maximal in pre-identified GABAergic and excitatory neurons in lamina II. The mean conductance ratio value for the populations of GABAergic neurons tested was 0.21 ± 0.03 (n = 12), intermediate between the values for NR2A/B and NR2C/D. Figure 1B also shows that the mean voltage of maximal inward current (VMIC) is -48 ± 2 mV (n = 12), again an intermediate value. These data suggest that NMDA receptors with both types of NR2 subunits are present on these EGFP+ lamina II neurons. In contrast, EGFP- neurons, mostly excitatory interneurons, had a significantly lower g(-90 mV)/g(MIC) ratio (0.15 ± 0.01 vs 0.21 ± 0.03, n = 11, p < 0.05) than EGFP+ neurons. Consistent with this difference in conductance ratio, average VMIC of EGFP- neurons is significantly more positive than in EGFP+ neurons (-43 ± 2 mV vs - 48 ± 2 mV, n = 11, p < 0.05), indicating that EGFP- neurons dominantly express a higher percentage of NR2A/B type NMDA receptors and less NR2C/D than EGFP+ cells. These data show that NMDA receptor composition in the two interneuronal populations of lamina II are different.

### Pharmacological test of NR2 subunit confirms the expression of NR2A/B and NR2C/D type NMDA receptors

Using pharmacological analysis, we demonstrated that conductance ratio and VMIC are good indicators of NMDA receptor subtypes expressed by dorsal horn interneurons. When NMDA was co-applied with UBP141(25 μM), a mildly selective NR2C/D antagonist [20], to an EGFP+ neuron, the current-voltage relationship developed a smaller conductance ratio and less negative VMIC (Figure 2A and 2B). Figures 2C and 2D show the individual and mean values of g(-90 mV)/g(MIC) ratio and VMIC recorded from EGFP+ neurons. Mean conductance ratio significantly changed from 0.19 ± 0.03 to 0.08 ± 0.02 (n = 5, p < 0.05, paired t-test), while VMIC shifted from -47 ± 3 mV to -39 ± 3 mV (n = 5, p < 0.05, paired t-test) after co-application of UBP141 and NMDA. Both parameters got significantly closer to those of NR2A/B type NMDA receptors. This suggests that in the presence of UBP141, most of the NR2C and NR2D containing NMDA receptors are blocked and that NR2A/B type NMDA receptors dominate the current.

When NMDA was co-applied with EAB318 (100 nM), an NR2A/B selective blocker [21], to an EGFP+ neuron, the current voltage relationship shifted to a larger g(-90 mV)/g(MIC) ratio and a more negative VMIC (Figure 3A and 3B). As shown in Figure 3C and 3D, the individual and mean values of g(-90 mV)/g(MIC) ratio and VMIC became NR2C/D type after co-application of EAB-318 and NMDA. Specifically, the mean conductance ratio changed significantly from 0.20 ± 0.03 to 0.41 ± 0.05 (n = 6, p < 0.05, paired t-test), while VMIC shifted from -48 ± 2 mV to -56 ± 3 mV (n = 6, p < 0.05, paired t-test) This suggests that mainly NR2C/D containing NMDA receptors remained after blocking NR2A/B containing NMDA receptors. These results support our interpretation that both types of NR2 subunits, NR2A/B and NR2C/D are present on inhibitory interneurons of lamina II.

The data shown in Figure 1 indicate that EGFP- neurons mainly express NR2A/B containing NMDA receptors. Under these conditions, selective blocking of NR2A/B type subunits is not expected to cause a significant shift towards NR2C/D like responses. To confirm this speculation, we investigated the effect of EAB318 and UBP 141 on the conductance ratio and VMIC of EGFP- neurons. The results are shown in Figure 4. Co-application of UBP141(25 μM) did not significantly change (-90 mV)/g(MIC) (0.10 ± 0.01 vs 0.15 ± 0.01; n = 6, p = 0.09, paired t-test) or VMIC (-38 ± 2 mV vs - 43 ± 3 mV; n = 6, p = 0.14, paired t-test). Similarly, EAB318 (100nM) failed to significantly alter values for g(-90 mV)/g(MIC) (0.18 ± 0.03 vs 0.14 ± 0.02; n = 7, p = 0.26, paired t-test) or VMIC (-42 ± 2 mV vs - 44 ± 2 mV; n = 7, p = 0.59, paired t-test). These results support our speculation that EGFP- cells predominantly express NR2A/B type NMDA receptors.

### Synaptic NMDA receptors on EGFP+ and EGFP-

NMDA application activates receptors at both synaptic and extrasynaptic sites. To identify synaptic NMDA receptors, we used focal synaptic stimulation in the presence of AMPA, GABA_A _and glycine receptor antagonists (NBQX, SR95531, strychnine respectively). Extracellular Mg^2+ ^concentration was kept at 100 μM to keep conditions comparable to those used in the experiments with exogenous NMDA application.

NMDA receptor-mediated, monosynaptic EPSCs were studied following addition of NBQX (see Methods) after first identifying monosynaptic AMPA EPSCs associated with focal stimulation at 10 Hz. In order to obtain the current voltage relationships for these NMDA EPSCs, we stepped the membrane to different holding potentials from -90 mV to +50 mV at 20 mV increments and then focally stimulated. After subtracting baseline membrane currents to isolate the NMDA EPSCs (Figure 5A), we measured EPSC amplitude 10 ms after the initial AMPA EPSC peak at each holding potential (see Methods). These amplitudes were plotted as a function of voltage, allowing us to again measure the conductance ratio of the NMDA receptor subtypes, but this time at subsynaptic regions. The NMDA EPSCs had an averaged reversal potential of 2.2 ± 0.6 mV, n = 37, close to the reversal potential of NMDA receptors, indicating good space clamp of these synapses (example in Figure 5B). The g(-90 mV)/g(MIC) ratio of synaptic NMDA EPSCs from EGFP+ and EGFP- neurons were calculated and compared. As shown in Figure 5C, the ratios of both subpopulations of interneurons were close to those of NMDA receptors with high Mg^2+ ^sensitivity, suggesting that mainly NR2A/B, not NR2C/D subunits, are synaptically expressed on both EGFP+ (0.09 ± 0.01, n = 18) and EGFP- neurons (0.09 ± 0.01, n = 19).

**Figure 6:**
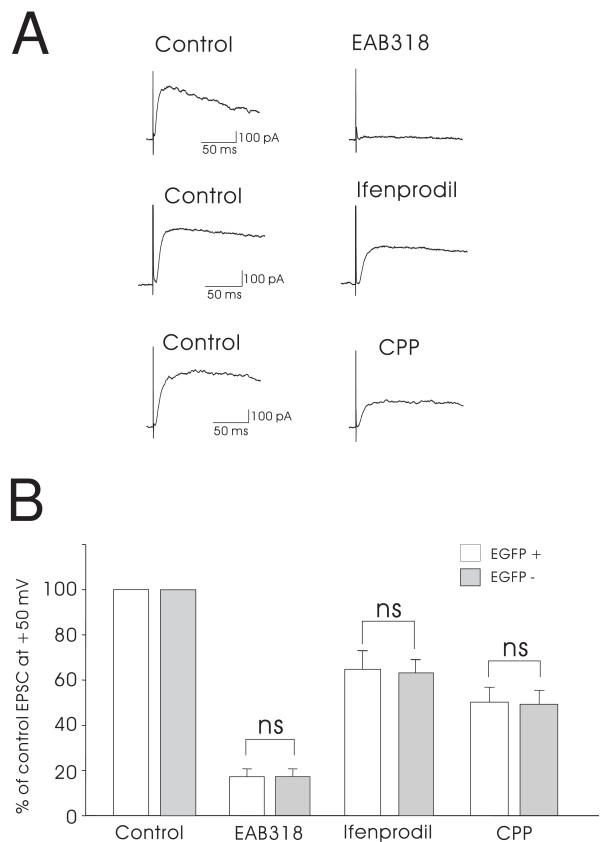
**Effect of NR2A and NR2B antagonists on synaptic NMDA receptors**. A, Representative traces show the inhibiting effects of EAB-318 (200 nM), ifenprodil (3 μM) and CPP (200 nM) on NMDA EPSCs evoked by focal stimulation and recorded from 3 EGFP+ neurons. Cells were held at +50 mV while EPSCs were recorded. B, shows the summary of NMDA receptor pharmacology recorded from EGFP+ and EGFP- neurons. The percentages of inhibition by EAB318, ifenprodil, and CPP on synaptic NMDA EPSCs were not significantly different between EGFP+ and EGFP- neurons (ns; not significant). We were able to obtain washout data on several of these neurons. The washout of antagonists ranged from 25% to 100% recovery of the blocked portion of the original NMDA EPSC amplitude. For the EGFP+ neurons, we were able to hold the neuron under study long enough to show washout in 6/16 neurons exposed to EAB318, 2/11 to CPP and 1/8 to ifenprodil. For the EGFP- neurons, we recorded partial to full recovery from 5/20 neurons exposed to EAB318, 2/14 neurons exposed to CPP and 2/8 neurons exposed to ifenprodil.

We further tested NR2 subunit composition of synaptic NMDA receptors for both EGFP+ and EGFP- neurons using a pharmacological approach. With the membrane potential held at +50 mV, focal stimulation was applied at 0.06 Hz in the presence of NBQX, SR95531 and strychnine. As shown in Figure 6A and 6B, EAB318 (200 nM), the antagonist for NMDA receptors with NR2A/B subunits [21], depressed the amplitude of NMDA EPSCs to 17.4 ± 3.5% (n = 16, p < 0.05, paired t-test) of the control amplitude for EGFP+ neurons and 17.5 ± 3.4% (n = 20, p < 0.05, paired t-test) for EGFP- neurons. This strong block confirms that both EGFP+ and EGFP- neurons expressed mainly NMDA receptors with NR2A/B subunits at synapses.

Ifenprodil (3 μM), a selective NR2B antagonist [22], and CPP, an NR2A preferring antagonist [23], were also tested by superfusion onto slices. Ifenprodil was superfused for 25 minutes with continuous focal stimulation at 0.05 Hz to allow for activity-dependent blockade of NMDA receptors. Before each stimulus, holding potential was stepped from -70 mV to +50 mV for 1 sec, allowing the membrane potential to remain at negative values most of the time. Under these conditions, ifenprodil decreased the amplitude of NMDA EPSC to 64.4 ± 8.3% (n = 8, p < 0.05, paired t-test) for EGFP+ neurons and to 63.5 ± 6.0% (n = 8, p < 0.05, paired t-test) for EGFP- neurons (Figure 6A and 6B). At a concentration of 200 nM, CPP diminished NMDA EPSC amplitudes to 50.2 ± 6.7% (n = 11, p < 0.05, paired t-test) for EGFP+ neurons and to 49.2 ± 6.3% (n = 14, p < 0.05, paired t-test) for EGFP- neurons (Figure 6A and 6B). The inhibition caused by each antagonist tested was not significantly different between EGFP+ and EGFP- neurons (Figure 6B). We were able to obtain partial to full washout in some of these recordings (Additional File 1). These results, together with the conductance ratio measurements (Figure 5C), suggest that NR2A and NR2B subunits are the dominant receptor subunits mediating NMDA EPSCs on both EGFP+ and EGFP- neurons.

## Discussion

We have identified NMDA receptor subtypes expressed by two different populations of lamina II interneurons in the spinal cord of GAD67 EGFP knock-in mice. The GABAergic EGFP+ neurons express both NR2A/B and NR2C/D subunit containing NMDA receptors. The mostly excitatory, EGFP- neurons mainly express NR2A/B containing receptors with minimal evidence for NR2C/D subunit expression. Furthermore, we found evidence that only NR2A and NR2B subunits are expressed as synaptic NMDA receptors in nearly identical proportions on both inhibitory and excitatory interneurons.

### Concurrence of NMDA subunit analysis by conductance ratio, voltage of maximal inward current and pharmacology

Without antagonists that are perfectly subunit selective, it is often difficult to use pharmacology to identify NMDA receptor subunit composition by quantifying drug-induced changes in current amplitude. For example, UBP-141 is only mildly selective for NR2C/D subunit mediated responses and can also block NR2A/B subunit mediated responses. Therefore a depression of current amplitude does not provide information about the subunit contribution to the receptors mediating NMDA evoked current responses. Instead, we quantified the voltage dependent Mg^2+ ^sensitivity of the NMDA response by defining two electrophysiological measures, conductance ratio (g(-90 mV)/g(MIC)) and VMIC, and compared our values to those from heterologous expression data [11,19]. Both conductance ratio and VMIC values determined for NMDA induced currents recorded from EGFP+ neurons were between those of NR2A/B and NR2C/D subunits suggesting that both types of subunits exist on these GABAergic neurons. Consistent with this observation, the NR2C/D preferring antagonist, UBP-141, shifted these values towards those of NR2A/B containing receptors. Conversely, the NR2A/B preferring antagonist, EAB318, shifted these values towards those of NR2C/D type receptors. Thus interpretation of conductance ratio and VMIC values was supported by the actions of subunit selective antagonists in a manner that is independent of current amplitude.

### Synaptic NMDA receptors

As is the case for NMDA currents studied using whole cell agonist application, synaptic currents mediated by NMDA receptors are difficult to analyze for subunit composition due to lack of highly subunit specific antagonists. Predictions made from focally stimulated NMDA EPSCs for unidentified lamina I and II neurons in neonatal rats indicated that synapses co-expressing AMPA and NMDA receptors have both NR2A and NR2B subunits while silent synapses have mainly NR2A subunits [24]. These predictions were based on NMDA receptor mediated EPSC decay kinetics. Another study used NMDA EPSC decay kinetics and lack of effect of ifenprodil to conclude that NR2A subunits predominate at synapses between primary afferent terminals and lamina II neurons in the adult rat [12]. Using conductance ratio to assess NR2 subunit expression by mouse lamina II GABAergic and excitatory neurons, we found that all synapses tested were mediated by NMDA receptors with NR2A/B subunits.

To probe more specifically for NR2A and B subunit contributions to synaptic receptors, we tested the sensitivity of NMDA EPSCs to three antagonists; the NR2A/B antagonist EAB318, the NR2A preferring antagonist, CPP and the NR2B antagonist, ifenprodil. CPP and ifenprodil both produced partial blocks of NMDA EPSCs from both populations of neurons. These effects indicate that both NR2A and NR2B are expressed at these synaptic sites in lamina II. EAB318 used at concentrations that block both NR2A and B decreased the amplitude of NMDA EPSCs by a little over 80%, indicating NR2A/B expression dominates postsynaptically. This also confirmed our earlier observations on unidentified rat lamina I and II neonatal rat neurons [24]. However, the absence of complete block of the NMDA EPSCs by EAB-318 suggests that a small amount of synaptic current may be carried by NR2C/D NMDA receptor subunits. However, if this is true, the presence of these subunits was too small to be detected as a significant contributor to conductance ratio in the excitatory interneurons tested here.

It is also interesting to consider why our observations differ from earlier studies [12] concluding that NR2A predominates at synapses between primary afferent terminals and dorsal horn neurons. Multiple reasons may contribute to this difference. First, in the current study, we evoked NMDA EPSCs by focal stimulation that can activate presynaptic excitatory interneurons and also primary afferent axons. Therefore focal stimulation samples a much wider range of excitatory inputs to the neuron under study than dorsal root stimulation. Second, we used juvenile mice (P14-P21) and NR2 subunit expression might be different from that of adult rats. During postnatal development, NR2B is replaced by other NR2 subunits [10]. Therefore, it is possible that NR2B subunits are expressed in lamina II interneurons at early stages of development and are replaced by NR2A receptors during maturation. Third, the NR2 composition of NMDA receptors on mouse lamina II neurons might be different from that of rats. Finally, a lack of response to ifendprodil was used to conclude that NR2B was not present postsynaptically [12]. However, it is possible that the application time was too short for the activity dependent block by ifenprodil [25]. We found that the maximal blocking effect of 3 μM ifenprodil required up to 20 minutes of perfusion in our spinal cord slice preparation. Overall, our data show that on average, NMDA receptors expressed postsynaptically on lamina II inhibitory and excitatory neurons in P14-P21 mice are composed primarily of NR2A and NR2B subunits.

### Synaptic NMDA and extrasynaptic NMDA receptors expressed on lamina II interneurons

In our study, bath application of NMDA activates synaptic and extrasynaptic receptors on the inhibitory and excitatory neurons under study. While the dominant synaptic receptors are NR2A/B type receptors, our bath application data indicate that NR2C/D receptors as well as NR2A/B receptors are expressed by inhibitory neurons and these may be extrasynaptic. We did not see significant evidence for NR2C/D subunit expression by excitatory interneurons. In situ hybridization studies report the distinct existence of NR2A and NR2B subunit mRNA in rodent lamina II [26-28]. mRNA of NR2D, but not NR2C, is also expressed in lamina II [27]. Our experiments using conductance ratio and pharmacology cannot distinguish between NR2C and NR2D subunits. However, based on the absence of NR2C mRNA detection as mentioned above, it is likely that NR2D is the main contributor when our data showed high conductance ratio or EAB-318 insensitivity. Consistent with these findings, Momiyama [12] reports the existence of NR2D-like single channel conductance at extrasynaptic regions in lamina II of the adult rats.

### Functional implications of NR2 subunits expressed on excitatory interneurons

The expression pattern of NR2 subunits observed for inhibitory neurons, which includes both NR2A/B and NR2D, is similar to that observed for the larger NK1R+, excitatory projection neurons in lamina I [11]. In contrast to those two populations of dorsal horn neurons, the conductance ratio and VMIC values found for EGFP- neurons only had characteristics of NR2A/B subunits and were not significantly altered by EAB-318 or UBP-141. Therefore, in EGFP- neurons, which are predominantly excitatory interneurons, NMDA receptor characteristics are less heterogeneous.

Disinhibition-induced recruitment of polysynaptic Aβ input onto lamina I projection neurons is relayed by excitatory interneurons. This recruitment is blocked by the non-selective NMDA receptor antagonist D-APV [6]. Here we show that this pathway might be mediated by NR2A/B containing NMDA receptors because excitatory input onto excitatory interneurons is mediated by receptors containing these subunits. This suggests that selective inhibition of NR2A/B containing receptors would be effective at reversing pain under conditions of disinhibition.

### Functional implications of NR2 subunits expressed on inhibitory interneurons

Our results suggest that NR2D containing NMDA receptors are likely to be expressed at extrasynaptic regions on inhibitory interneurons. NR2D containing receptors have a much higher affinity for glutamate [29], are less sensitive to voltage-dependent Mg^2+ ^block [9,19] and have a much longer open time than NR2A/B containing receptors [10]. Thus, inhibitory interneurons, which are expressing more extrasynaptic NR2D subunits, might be more sensitive to ambient glutamate in the extrasynaptic space than excitatory interneurons and might allow more Ca^2+ ^influx. Accumulation of ambient glutamate occurs due to spillover associated with high neuronal activity, glial release [30,31] and downregulation of glutamate transporters [32,33]. Under mildly stressful conditions, ambient glutamate may selectively activate inhibitory interneurons through extrasynaptic NMDA receptors with NR2D subunits and enhance inhibition of pain signaling. Under very stressful conditions of peripheral or central injury or inflammation, sustained elevation of glutamate could lead to selective death of inhibitory interneurons and disinhibition could occur. A report of NMDA receptor dependent cell death in the superficial dorsal horn in a model of neuropathic pain supports this possibility [34]. Selective apoptosis of GABAergic dorsal horn interneurons has been reported after nerve injury [35-37] although there are conflicting reports on changes in GABAergic interneurons [38]. Thus, there might be a system in which ambient glutamate selectively activates inhibitory interneurons through NR2D containing NMDA receptor with physiological or pathological consequences.

In conclusion, we demonstrated that NR2A and NR2B containing receptors with high Mg^2+ ^sensitivity and NR2D containing NMDA receptors with low Mg^2+ ^sensitive are both expressed on GABAergic lamina II interneurons. Lamina II excitatory interneurons express predominantly NMDA receptors with NR2A/B subunits. The differential expression of NR2D containing NMDA receptors on lamina II inhibitory interneurons compared to excitatory interneurons suggests a role for NR2D containing NMDA receptors in modulating the activity of inhibitory interneurons.

## Method

### Ethical approval

All experiments were conducted with the approval of the Columbia University Institutional Animal Care and Use Committee and in accord with the Guide for the Care and Use of Laboratory Animals. Lumbar spinal cords were obtained from mice of postnatal day 14 (P14) to P21. Animals were first anesthetized with isoflurane and then decapitated.

### Transverse slice preparation

Mice used in this study were homozygous for a transgene that has enhanced green fluorescent protein (EGFP) expression driven by the mouse *gad1 *gene promoter. This gene encodes for GAD67 [39], a 67 kDa isoform of the enzyme required to produce GABA [40,41]. Breeding pairs of these mice were obtained from The Jackson Laboratory and interbred at our facility. Nearly all of the fluorescent neurons are GABAergic in lamina I and II of the spinal cord [15,16,39].

Spinal cords were excised and placed in ice-cold dissection Krebs solution (in mM: 95 NaCl, 2.5 KCl, 26 NaHCO_3_, 1.25 NaH_2_PO_4_H_2_O, 6 MgCl_2_, 1.5 CaCl_2_, 25 glucose, 50 sucrose, 1 kynurenic acid), saturated with 95%O_2_/5%CO_2_. After removal of the dura mater and arachnoid membranes, all dorsal and ventral roots were cut close to the cord and the spinal cord embedded in low melting agarose (Invitrogen Life Technologies) for slicing. Transverse slices (400 μm) were obtained using a Leica VT1200S vibrating blade microtome. Slices were then incubated in 95%O_2_/5%CO_2 _saturated recovery Krebs (same as dissection Krebs, but lacking kynurenic acid) at 37°C for 1 h and then at room temperature until use.

### Recording from pre-identified EGFP+ and EGFP- lamina II neurons

Dorsal horn neurons were viewed using an upright microscope with a 40x water-immersion objective, infrared differential interference contrast (IR-DIC) and fluorescence. Whole-cell patch-clamp recordings were made from GABAergic EGFP+ and mostly excitatory EGFP- neurons. EGFP+ neurons were initially identified under epi-fluorescence then approached with a pipette using DIC optics. EGFP- neurons were indentified as non-fluorescent under epi-fluoresence then patched under DIC. Intracellular solution used for most of these experiments had the following composition (in mM): Cs-methylsulfonate 130, Na-methylsulfonate 10, EGTA 10, CaCl_2 _1, HEPES 10, QX-314·Cl 5, Mg^2+^-ATP 2, pH adjusted to 7.2 with CsOH, osmolarity adjusted to 290 with sucrose. Junction potentials were measured empirically and corrected in the bath before GOhm seal formation onto each cell.

For the extracellular bath, modified Krebs solutions were used. To prevent possible neurotoxicity associated with Ca^2+ ^influx through activated NMDA receptors, we replaced 95-98% of the extracellular Ca^2+ ^with 3 mM Ba^2+ ^for experiments in which NMDA was bath applied. The barium Krebs comprised: NaCl 125, KCl 2.5, NaH_2_PO_4_1.25, NaHCO_3 _26, glucose 25, MgCl_2 _0.1, CaCl_2 _0.1, BaCl_2 _3 and pH 7.4. TTX (0.5 μM), SR95531 (10 μM) and strychnine (1 μM) were included in the extracellular solutions to eliminate action potential generation and involvement of inhibitory circuits. Krebs solution for synaptic studies comprised NaCl 125, KCl 2.5, NaH_2_PO_4_1.25, NaHCO_3 _26, glucose 25, MgCl_2 _0.1, CaCl_2 _2 and pH 7.4. SR95531(10 μM) and Strychnine (1 μM) were included in the extracellular solutions to block inhibitory circuits.

### Analysis of NMDA induced membrane currents

To obtain the current-voltage relationships of NMDA receptor-mediated currents, NMDA (15 μM) was superfused onto pre-identified, lamina II neurons for 4-5 minutes following several minutes of baseline, whole-cell recording. Triangle voltage ramp commands (the ramp up and ramp down were 0.9 sec duration each) were applied continuously at low frequency (0.05 Hz). Digital sampling frequency was 10 KHz. NMDA applications were repeated 2 -3 times before NMDA co-application with antagonists. Current responses to triangle voltage ramps before and after recovery from NMDA application were averaged as a control current then subtracted from each triangle ramp made during NMDA induced currents. The resulting NMDA current ramps were plotted as a function of membrane potential and further analyzed. The current responses to voltage ramps before and after the first NMDA application were generally changing as a function of time. By the time of the second NMDA application, current responses to control ramps before and after NMDA application were similar. Thus the data for the first NMDA application were not included in this report. To minimize noise for measuring the following parameters, NMDA current ramps were subjected to a rolling average procedure over a 100 msec time frame (not shown).

For each voltage ramp during NMDA applications, the membrane current at -90 mV (I(-90 mV)), the maximal inward current (MIC), and the membrane potential for the MIC (VMIC) were measured. The current measured at -90 mV holding potential was determined as I(-90 mV). The MIC was initially determined as the most negative current value in the rolling average. The VMIC was then determined as the voltage corresponding to the MIC. Because each ramp had an up and a down phase, each parameter from a ramp current had a pair of values and they were averaged for subsequent analysis. The conductance at -90 mV and MIC (g(-90 mV) and g(MIC) respectively) as well as conductance ratio were then calculated based on the formulae:

To compare NMDA receptor g(-90 mV)/g(MIC) ratios, conductance ratios, under different pharmacological conditions, we averaged three ratio values calculated for each NMDA application near the peak NMDA response at -70 mV. The ratios under different pharmacological conditions or represented by different neuron populations were then compared.

Similar to the previous paper [11], only cells starting with reversible NMDA induced membrane currents, in which the difference between g(-90 mV)/g(MIC) ratios during wash-in and wash-out of NMDA was less than 0.15, were included for analysis. Cells with high membrane holding current at -70 mV (inward currents larger than -100 pA) were discarded.

### Analysis of NMDA excitatory postsynaptic current (EPSC)

Recorded cells were held at -70 mV under voltage clamp control unless otherwise specified. Focal synaptic stimulation was applied through a theta pipette filled with bath solution. The pipette was placed about 50 μm from the recorded cell. Stimulus intensities ranged from 30 V to 80 V and each stimulus was presented for 100 μs. Evoked EPSCs (eEPSCs) were tested at 10 Hz to ensure that each excitatory response included in the analysis was monosynaptic. The time from the stimulus artifact to the peak of the monosynaptic AMPA receptor mediated EPSCs (AMPA EPSCs) was measured from events recorded at 10 Hz stimulation. Subsequently, 5 μM NBQX, 1 μM strychnine, and 10 μM SR95531 was added to the bath and NMDA synaptic currents were stimulated at 0.06 Hz while recording at different membrane potentials from -90 mV to +50 mV using 20 mV steps. The amplitudes of monosynaptic NMDA receptor mediated EPSCs (NMDA EPSCs) were measured 10 ms after the peak of the monosynaptic AMPA EPSC to ensure minimal contamination by polysynaptic NMDA receptors (McBain and Mayer, 1994).

## Materials

SR 95531 hydrobromide were purchased from Tocris Cookson (Bristol, UK). QX-314·Cl was purchased from Alomone labs (Jerusalem, Israel). Strychnine was obtained from Sigma-Aldrich (St Louis, USA). NBQX and Ifenprodil were purchased from Ascent (Princeton, USA). EAB318 was provided by Wyeth Research. EAB318 has IC50 of 20, 80 and 3500 nM for NMDA receptors with NR2A, NR2B and NR2C respectively [21]. UBP141 was synthesized as described [20]. The K_i _of UBP141 for NMDA receptors with NR2A - NR2D are 14, 19, 4 and 2.7 μM respectively [20].

## Competing interests

The authors declare that they have no competing interests.

## Authors' contributions

All of the authors contributed to conception and design of the project, analysis and interpretation of data, drafting the article, revising it critically for important intellectual content, and approval of the final version. HS prepared and recorded from the spinal cord slices. All experiments were performed at Columbia University Medical Center, New York, NY, USA.

## Supplementary Material

Additional File 1 **Representative traces showing inhibition of focally evoked NMDA EPSCs by EAB-318 (200 nM), ifenprodil (3 μM) and CPP (200 nM) followed by recovery of NMDA EPSC amplitudes during antagonist washout. **The upper and the bottom data are from different EGFP+ neurons. Middle traces are from an EGFP- neuron. Click here for file
