# Supplementary figures and images for: NR2 subunits and NMDA receptors on lamina II inhibitory and excitatory interneurons of the mouse dorsal horn
Source: Mol Pain. 2010 May 6;6:26. doi: 10.1186/1744-8069-6-26 (PMC2879240; doi:10.1186/1744-8069-6-26)

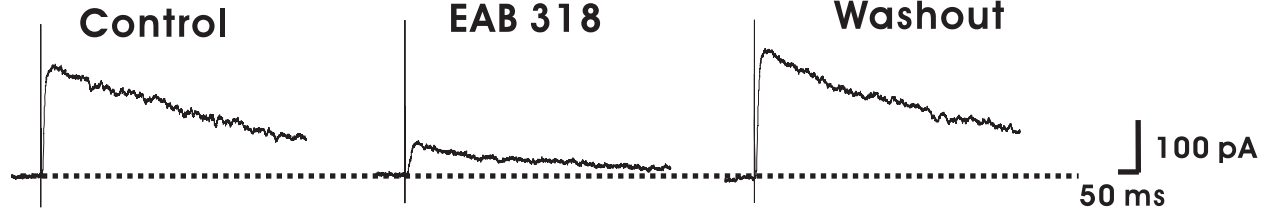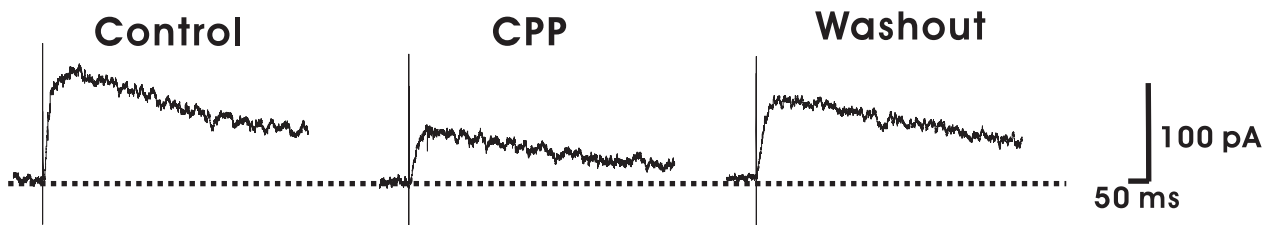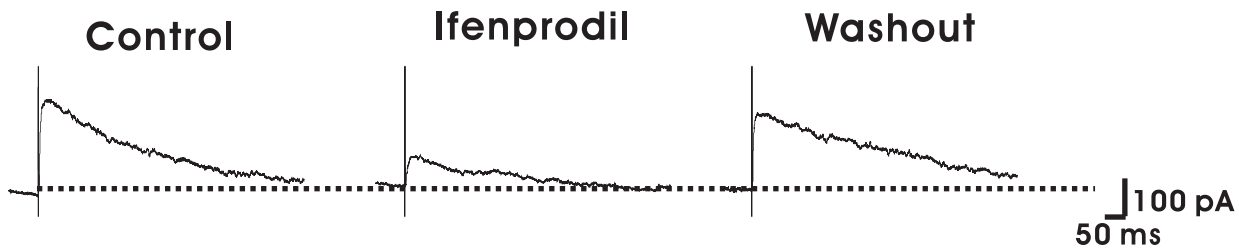

Supplement: Additional File 1 — Representative traces showing inhibition of focally evoked NMDA EPSCs by EAB-318 (200 nM), ifenprodil (3 μM) and CPP (200 nM) followed by recovery of NMDA EPSC amplitudes during antagonist washout. The upper and the bottom data are from different EGFP+ neurons. Middle traces are from an EGFP- neuron. [file 1744-8069-6-26-S1.PDF]
